# Temporal Association between Hampton’s Hump Pulmonary Embolism and First-Dose ChAdOx1 nCov-19 Vaccine in a Patient with Activated Protein C Resistance

**DOI:** 10.3390/vaccines10101659

**Published:** 2022-10-03

**Authors:** Gernot Kriegshäuser, Andreas Braunsteiner

**Affiliations:** 1 IHR LABOR Medical Diagnostic Laboratories, Wagramer Straße 144, 1220 Vienna, Austria; 2 School of Management, University of Applied Sciences BFI, 1020 Vienna, Austria; 3 Radiologicum Wieden, 1040 Vienna, Austria

**Keywords:** temporal association, pulmonary embolism, APC resistance, ChAdOx1 nCov-19, factor V_Leiden_

## Abstract

A 58-year-old man presented to his practitioner with right-sided pleuritic chest pain, dyspnea, and fatigue 18 days following the first dose of the ChAdOx1 nCov-19 vaccine. Chest radiography showed a basal wedge-shaped consolidation indicative of a Hampton’s hump in the right lower lobe, which was confirmed by subsequent computed tomography pulmonary angiography. The major laboratory abnormalities were a markedly elevated D-dimer level of 7.53 µg/mL (normal range < 0.5 µg/mL), a CRP level of 62.8 mg/L (normal range < 5 mg/L), and a previously unknown activated protein C resistance of 1.3 (normal range ≥ 1.8). Genetic analysis identified the patient to be heterozygous for the FV_Leiden_ mutation. Neither other errors of hemostasis nor antibodies against platelet factor 4-polyanion complexes could be observed. Moreover, we failed to demonstrate Severe Acute Respiratory Syndrome Coronavirus-2 infection. The patient fully recovered with no sequelae but is being continued on long-term anticoagulation given his risk of recurrent venous thromboembolism. Here we report on the temporal association between the first dose of the ChAdOx1 nCov-19 vaccine and extensive pulmonary embolism in an otherwise healthy patient with activated protein C resistance, however, further investigation is needed to prove causality.

## 1. Introduction

The ongoing coronavirus disease 2019 (COVID-19) pandemic has devastated economies and caused unprecedented challenges to healthcare and food systems around the world. In March 2021, vaccination using the ChAdOx1 nCov-19 (AZD1222) vaccine (Oxford-AstraZeneca) was paused in several European countries because of unusual thrombotic events observed after vaccine administration, sharing distinct clinical features (thrombosis in uncommon locations, thrombocytopenia, predominantly observed in young adults and women) [1,2]. Subsequently, vaccine-induced immune thrombotic thrombocytopenia (VITT), a variant of heparin-induced thrombocytopenia (HIT), was identified as a cause of thrombosis in these patients [3]. HIT is a progressive thrombotic condition that can cause both venous and arterial thrombosis, typically 5 to 14 days after exposure to heparin. It is more common in female patients, particularly those who receive unfractionated heparin, and the diagnosis is confirmed by the presence of platelet factor 4-polyanion complexes (PF4) antibodies [3]. Moreover, other severe side effects thromboses combined with immunothrombocytopenia (ITP) were reported after the administration of the ChAdOx1 nCov-19 vaccine [4].

Proteolytic inactivation of factors Va (FVa) andVIIIa (FVIIIa) by activated protein C (APC) and its cofactors protein S and factor V (FV) is a key process in the physiological down-regulation of blood coagulation [5]. A poor response of plasma to exogenous APC, better known as APC resistance, was first discovered in selected thrombophilic families [6] but was soon recognized as the most frequent cause of inherited thrombophilia [5,7]. In most cases, APC resistance is caused by a single point mutation in the factor V gene (FV_Leiden_, Leiden mutation, G1691A). The FV_Leiden_ mutation is present in about 5% of the general population and predisposes them to venous thromboembolism (VTE^)^ [5,8]. The risk of VTE is increased approximately 7-fold and 80-fold in heterozygous and homozygous carriers, respectively [9]. Most of the FV_Leiden_ carriers never experience VTE, however, additional risk factors (e.g., protein C or protein S deficiency, oral contraceptive use, trauma, surgery, pregnancy) may be identified in those patients who develop VTE [5,10].

Here, we report on the radiological and laboratory diagnostic workup of an otherwise healthy 58-year-old man with extensive pulmonary embolism (PE) 18 days following the first dose of the ChAdOx1 nCov-19 vaccine.

## 2. Case

A 58-year-old man presented to his practitioner with right-sided pleuritic chest pain, dyspnea, and fatigue 18 days following the first dose of the ChAdOx1 nCov-19 vaccine. At the time of anamnesis, we could not identify risk factors for VTE (e.g., malignant, autoimmune, or infectious disease), however, chest auscultation indicated a reduced air entry over the right lower quadrant.

**Figure 1:**
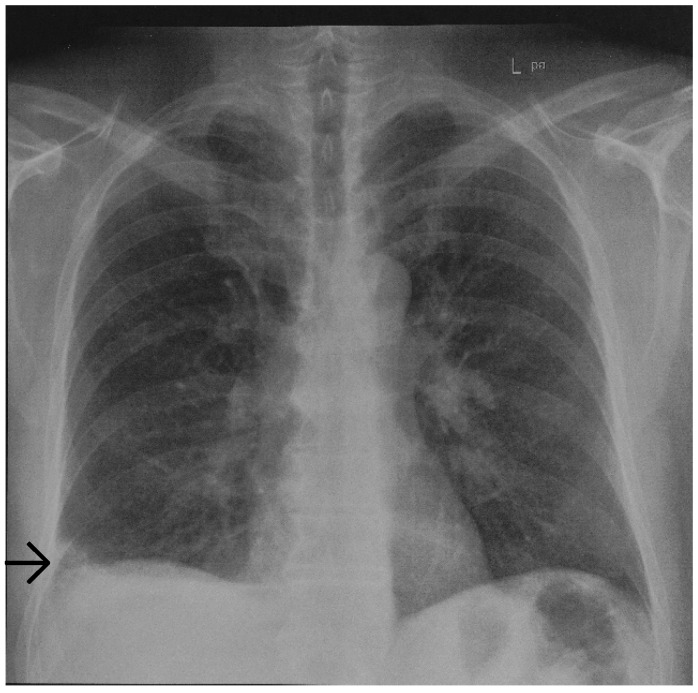
Posterior-anterior view of a chest radiograph demonstrating a basal wedge-shaped consolidation indicative of a Hampton’s hump (arrow) in the right lower lobe.

**Figure 2:**
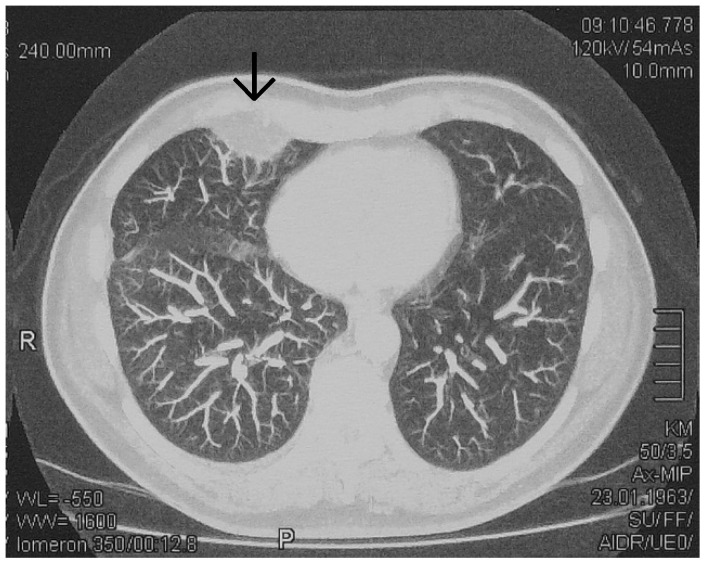
Computed tomography pulmonary angiogram (CTPA) showing an area of subpleural PI (arrow) in the right lower lobe.

Initial chest radiography revealed a basal, wedged-shaped consolidation in the right lower lobe (Figure 1) which was concerning for a Hampton’s hump. This radiological sign was first described in 1940 by Hampton and Castleman, who performed an autopsy series to demonstrate the site of opacities seen on chest radiography in patients with PE compared with pulmonary infarction (PI) seen at autopsy [11,12]. PE is a common cause of PI; however, the true incidence of subsequent PI is variable. In patients diagnosed with PE, PI has been reported in 15% to 31% of patients on follow-up autopsy and in 9% to 36% of patients on computed tomography [13,14,15]. Hampton’s hump was demonstrated to be modestly specific for the diagnosis of PE but lacks sensitivity [16]. In a study evaluating radiographs of patients in the multicenter Prospective Investigation of Pulmonary Embolism Diagnosis (PIOPED) trial, Hampton’s hump showed a sensitivity of 22% and a specificity of 82% [17]. Therefore, computed tomography pulmonary angiography (CTPA) was performed showing an area of subpleural PI in the right lower lobe (Figure 2).

The only abnormal blood tests reported were a mild leukocytosis of 11.62 G/L (normal range 4.00–10.00 G/L), a mild thrombocytopenia of 143 G/L (normal range 150–350 G/L), a CRP and fibrinogen level of 62.8 mg/L (normal range < 5 mg/L) and 542 mg/dL (normal range 180–350 mg/dL), respectively, a markedly elevated D-dimer level of 7.53 µg/mL (normal range < 0.5 µg/mL), and a previously unknown APC resistance of 1.3 (normal range ≥ 1.8). Follow-up measurements six weeks after the initial examination revealed normalized blood cell counts with a negative CRP and a reduced D-dimer level of 1.73 µg/mL (normal range < 0.5 µg/mL).

Of note, antibodies against PF4 could not be observed, thereby ruling out VITT as a cause of PE. Moreover, antiphospholipid syndrome (APS) seems unlikely because the activated partial thromboplastin time (aPTT) was not found prolonged (24 s; normal range 23–32 s). Additionally, we observed normal levels of protein C, protein S, and antithrombin, and failed to demonstrate Severe Acute Respiratory Syndrome Coronavirus-2 (SARS-CoV-2) infection as indicated by repeatedly negative nasopharyngeal swab testing. Subsequent genetic analysis identified the patient to be heterozygous for the FV_Leiden_ mutation. The patient fully recovered with no sequelae but is being continued on long-term anticoagulation given his risk of recurrent VTE.

## 3. Discussion

Recently, a novel pathomechanism for the development of thrombosis after ChAdOx1 nCov-19 vaccination has been proposed [4]. Accordingly, splice variants of the SARS-CoV-19 Spike protein lacking their transmembrane anchor are shed into the bloodstream. Upon dissemination throughout the vascular system, secreted Spike variants could have the potential to induce severe side effects when binding to cells via the ACE2 receptor. These side effects may be explained by the formation of anti-Spike antibodies directed against Spike variants bound to the ACE2 receptor with the potential to result in antibody-dependent cell-mediated cytotoxicity (ADCC) and/or complement-dependent cytotoxicity (CDC)-mediated inflammatory reaction [4]. Moreover, other pathological reactions such as the neutrophil extracellular traps (NETs) host damage [18] or the capillary leak syndrome could potentially explain the side effects observed after ChAdOx1 nCov-19 vaccination [4,19].

In their population-based cohort study, Pottegård and colleagues [1] assessed the rates of cardiovascular and hemostatic events in the first 28 days after vaccination with the Oxford-AstraZeneca vaccine ChAdOx1-S in Denmark and Norway in order to compare them with rates observed in the general populations. Among 281,264 individuals, 59 venous thromboembolic events, including cerebral venous thrombosis, were observed in the vaccinated cohort compared with 30 expected based on the incidence rates in the general population. The authors, however, concluded, that the absolute risks of venous thromboembolic events were small, and their findings should be interpreted in the light of the proven beneficial effects of the vaccine, the context of the given country, and the limitations to the generalizability of the study findings.

While VITT-associated VTE has already been observed in a patient carrying the FV_Leiden_ mutation [20], this is, to the best of our knowledge, the first report on a patient heterozygous for FV_Leiden_ with PE and without the concomitant presence of anti-PF4 antibodies.

## 4. Conclusions

Here we report on the temporal association between the first dose of the ChAdOx1 nCov-19 vaccine and extensive PE in an otherwise healthy patient with APC resistance, however, further investigation is needed to prove causality. Nevertheless, it seems tempting to speculate that a secreted Spike variant or even anti-Spike antibodies synergized with FV_Leiden_ in our patient. Therefore, preemptive testing for APC resistance in individuals receiving ChAdOx1 nCov-19 vaccination could become a moot point.

## Data Availability

Not applicable.
